# Effect of Toothpaste on the Surface Roughness of the Resin-Contained CAD/CAM Dental Materials: A Systematic Review

**DOI:** 10.3390/jcm11030767

**Published:** 2022-01-31

**Authors:** Adolfo Di Fiore, Edoardo Stellini, Michele Basilicata, Patrizio Bollero, Carlo Monaco

**Affiliations:** 1 Department of Neuroscience, Section of Prosthetic and Digital Dentistry, University of Padova, 35122 Padova, Italy; edoardo.stellini@unipd.it; 2 Department of Systems Medicine, University of Rome Tor Vergata, 00133 Roma, Italy; michele.basilicata@ptvonline.it (M.B.); patrizio.bollero@ptvonline.it (P.B.); 3 Department of Surgery, Medicine, and Dentistry, University of Modena e Reggio Emilia, 41121 Modena, Italy; carlo.monaco@unimore.it

**Keywords:** CAD/CAM materials, toothbrushing wear, surface, roughness, surface integrity

## Abstract

Background: The purpose of this review is to describe the possible effect of toothbrushing on surface roughness of resin-contained CAD/CAM materials. Methods: Systematic literature search for articles published in peer-reviewed journals between January 2000 and February 2020 has been conducted, which evaluated the effect of brushing on surface roughness of resin-contained CAD/CAM dental materials. The research was conducted in Scopus, PubMed/Medline, Web of Science, Embase, and Science Direct using a combination of the following MeSH/Emtree terms: “brushing”, “resin-based”, “dental”, “CAD/CAM”, and “surface roughness”. Results: A total of 249 articles were found in the search during initial screening. Fifty-five articles were selected for the full-text evaluation after the steps of reading of abstract/title and remotion of duplicate. Only six articles fulfilled the inclusion criteria. The Cohen’s Kappa agreement test showed an index of 0.91 for full-text. Discussion: Four of five selected articles identified an increase of surface roughness on resin-contained CAD/CAM materials after toothbrushing. Although all the articles examined used different toothpastes with no homogeneous relative dentine abrasivity (RDA) and cycles of brushing, the findings are about the same. The possible reason is attributable to the compositions of the resin-contained CAD/CAM materials. Conclusions: The surface roughness of most resin-contained CAD/CAM materials was affected by artificial toothbrushing. Correct knowledge of the composition of the dental material and toothpastes is fundamental to avoid an increase of surface roughness on prosthetic rehabilitation.

## 1. Introduction

With growing awareness of esthetic rehabilitation, many patients require metal-free solutions [1]. Ceramic is the most used material for esthetic restorations in fixed prosthodontics. Surface roughness, translucency, resistance to wear, and mechanical properties are the main investigated characteristics of the ceramic surface [2]. In the last few years, computer-aided design/computer-aided manufacturing (CAD/CAM) has been introduced in the dentistry world and has improved the accuracy of prostheses, comfort for patients, and operative time [3,4]. Consequently, new different materials have been realized with different surfaces and mechanical behaviors [5,6]. The surface roughness is one of the factors that influenced the clinical survival of prosthetic rehabilitation, optical properties, wear of the antagonist teeth, and initiation of cracks [7]. Above the threshold Ra value of 0.2 μm for roughness, an increase of plaque accumulations has been observed on prosthetic materials [7]. The presence of bacteria on prosthetic rehabilitation becomes the main cause of biological complication, therefore, daily dental hygiene is necessary to remove plaque and prevent gingival inflammations [8]. Different factors influenced the surface roughness of the prosthetic materials, but the effect of brushing or polishing with toothpaste or prophylactic polishing pastes could be considered as one of the factors [9,10,11]. Regarding the polishing procedure by using the prophylactic pastes, several authors demonstrated the possible surface roughness alteration on prosthetic materials [10,11]. Few investigations on brushing are published [9]. However, most studies presented in the literature reported the abrasive effect of toothpaste and/or prophylactic pastes on the surface of composite materials and poly(methyl)methacrylate resin materials [12,13,14,15,16]. Commercially, resin-based CAD/CAM materials are used to produce prosthetic rehabilitation, moreover, different kinds of toothpastes are available with different relative dentine abrasivity (RDA indexes) [17]. However, few studies investigated the effect of brushing on these new materials. So, the purpose of this systematic review was to assess the effect of toothpaste on the surface roughness of the resin-contained CAD/CAM dental materials.

## 2. Materials and Methods

This systematic review was conducted in accordance with the Preferred Reporting Items for Systematic Reviews statement [18]. The PICO question was: “In the resin-contained CAD/CAM dental material (P), does the use of toothpaste (I) have any possible adverse effects (C) in terms of surface roughness modifications (O)?”.

### 2.1. Search Strategy

Electronic database searches of MEDLINE, EMBASE, Web of Science, Science Direct and Scopus were performed using the following keywords and MeSH/Emtree terms based on a search strategy used for searching MEDLINE: “brushing”, “resin-based”, “dental”, “CAD/CAM”, and “surface roughness”. In addition, a manual search of the bibliographies of the most relevant systematic reviews and of all included and excluded articles was employed to identify other eligible studies.

### 2.2. Screening Method and Data Extraction 

Titles and abstracts were screened, and the full texts of all potentially relevant publications were reviewed independently by the two authors (A.D.F., E.S.). Any disagreements between the two reviewers regarding inclusion were resolved by discussion. Cohen’s Kappa statistic was calculated after the full-texts examination. The investigators recorded the study title, authors, year of publication, journal in which the research was published, study type (in vitro or in vivo research), brushing procedure (i.e., toothpaste, RDA, timing), prosthetic materials investigated, and surface roughness values before and after brushing.

### 2.3. Inclusion and Exclusion Criteria 

The inclusion criteria were confined to full-text articles in English, published in peer-reviewed journals between 1 January 2000 and 28 February 2020, which evaluated the effect of brushing on surface roughness of resin-contained CAD/CAM dental materials. The exclusion criteria were articles that described other adverse effects than surface roughness, polishing procedure, letters to the editor, personal communications, reviews and meta-analyses. Surface roughness was the primary factor evaluated in each article. Subsequently, scientific articles that brought a better understanding of the different adverse effects of brushing on resin-contained CAD/CAM dental materials were identified to clarify and add knowledge.

### 2.4. Quality Assessment

The quality of each included study was individually evaluated following the Cochrane Collaboration guidelines. The Risk of Bias in Non-randomized Studies of Interventions (ROBINS-I) tool was utilized [19]. Each included study was classified as “low”, “moderate”, “serious” or “critical” risk of bias. Then, an overall score was given, judging the study at “low risk of bias” when it was assessed “low” in all domains, at “moderate risk of bias” when it was assessed “low” or “moderate” in all domains, at “serious risk of bias” when it was assessed “serious” in at least one domain or at “critical risk of bias” when it was assessed “critical” in at least one domain.

## 3. Results

**Figure 1:**
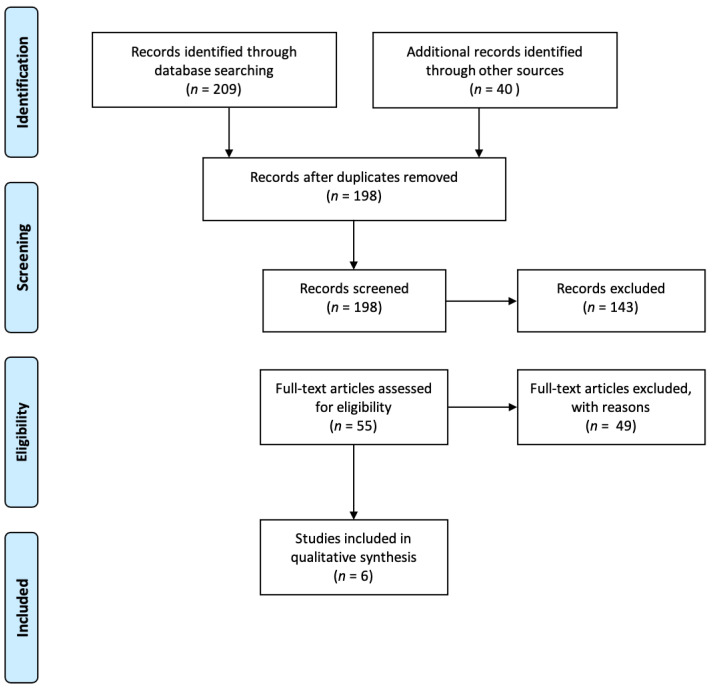
PRISMA flow chart of screened, withdrawn and included articles through the review process.

A total of 249 articles were found in the search during the initial screening. Two hundred and nine records were identified through database searching and 40 from the manual search. After duplicate studies had been removed, 198 records were screened. After title/abstract evaluation, 55 articles were selected for the full-text evaluation, of which six fulfilled the inclusion criteria (Figure 1). 

The main reasons for exclusion were that several studies investigated the effect of toothbrushing on direct composite and ceramic materials. The Cohen’s Kappa agreement test showed an index of 0.91 for full-text for the articles selected. Six articles were selected according to the inclusion criteria [20,21,22,23,24,25]. No clinical studies were included. All six articles investigated Lava Ultimate (3M Espe) and Vita Enamic (Vita Zahnfabrik). Three included the Cerasmart (GC, GC Europe NV) [21,23,24], two Gradia Block (GC) and Shofu Block Hc (Shofu) [21,23], one on Katana Avencia (Kuraray Noritake) [21], Paradigm MZ100 (3M ESPE) [22], Ambarino High Class (Creamed) [22], and Hybrid Resin Block (Yamamoto) [23]. All authors analyzed the surface roughness with a profilometer before and after the procedure of toothbrushing by using a toothbrush machine. Flury et al. [22] performed 3 measurements for specimen over a transverse length of Lt = 5.600 mm with a cut-off value of 0.8 mm and a stylus speed of 0.5 mm/second. Kamonkhantikul et al. [23] measured the sample tracing a length of 2 mm with a speed of 500 μm/s, and a cut-off length of 0.25. Five parallel measurements, each 400 μm apart, were performed perpendicular to the toothbrushing direction. Instead, Schmitt de Andrade et al. [24] made three profile measurements for each specimen for 4.2 mm along the specimen’s surface with a cut-off value of 0.25 mm and a stylus speed of 0.1 mm/s. No details on measurements with the profilometer were reported in the article of Morman et al. [20] and Koizumi et al. [21]. Nima et al. [25] used a 3D noncontact laser-scanning microscope to obtain the measurements and 3D images of the sample. All authors used the mean surface roughness (Ra) value in μm to compare the value before and after toothbrushing [20,21,22,23,24] except Nima et al. [25], who used the maximum relative depth (Rv). For Rv calculation, five measurements were made that started in the control area and extended into the brushed region.

Different toothpastes were used in the experiments. RDA index values were 70 for the toothpaste used by Flury et al. [22], 136 in the research of Koizumi et al. [21], 80 for Kamonkhantiku et al. [23], 70/80 for Schmitt de Andrade et al. [24], 44 for Nima et al. [25], and not identified in the article of Morman et al. [20]. Five of six selected articles identified an increase of surface roughness on resin-contained CAD/CAM materials after toothbrushing [21,22,23,24,25]. Some materials such as Cerasmart (GC) [21,22,23] and Shofu Block Hc (Shofu) [21,23] were more affected by toothbrushing than others such as Lava Ultimate (3M Espe) and Vita Enamic (Vita Zahnfabrik). Regarding the cycles of artificial toothbrushing, several frequencies were performed. Flury et al. [22] applied 3000 cycles that are equivalent to 6000 toothbrushing strokes. Koizumi et al. [21] applied the specimen to 20,000 reciprocal strokes (approximately 120 min), 40,000 cycles were applied by Kamonkhantiku et al. [23], and 1500 cycles for Morman et al. [20]. Instead, Schmitt de Andrade et al. [24] carried out 100,000 toothbrushing strokes and Nima et al. [25] submitted the sample to 300,000 toothbrushing strokes (150 cycles/min).

All data regarding authors, type of studies, toothbrushing test, and surface roughness analysis are reported in Table 1.

The risk of bias in six studies included was classified as moderate risk of bias (Table 2). Three studies [22,23,24,25] were deemed to have “low risk of bias” for selection of the major resin-contained CAD/CAM materials present in the dental market and for detailed description of the methods and results. The other three studies [20,21] were considered as “moderate risk of bias” due to the use of ceramic materials during the investigation and some missing data in the methodology used during the experimentations.

## 4. Discussion

The systematic review reported the relationship between toothpaste, RDA index, and surface roughness (Ra) for five articles [20,21,22,23,24] and maximum relative depth (Rv) for one [25] on resin-contained CAD/CAM dental materials. 

Flury et al. [22] investigated the effect of artificial toothbrushing on the CAD/CAM materials including different resin containing dental materials such as Lava Ultimate (3M ESPE), Vita Enamic (Vita Zahnfabrik), and Ambarino High-Class (Creamed). All the materials were stored in tap water in an incubator for 6 months at 37 °C. Each month all the samples were undergoing artificial toothbrushing for 500 cycles using a toothbrushing machine. The surfaces’ roughness was measured by using a profilometer before and after the procedures of storage and toothbrushing. The findings demonstrated different behaviors of the resin-contained CAD/CAM materials. The surface roughness (Ra) significantly increased after artificial toothbrushing and storage for Ambarino High-Class (Ra and Rz, *p* < 0.001). Instead, Lava Ultimate and Vita Enamic showed no significant change in surface roughness after artificial toothbrushing and storage compared with after polishing (*p* > 0.05). The reason could be explained by the different filling materials used to compose the blocks. The Ambarino High-Class presents a 70 weight % ceramic-like inorganic silicate glass filler particles and 30 weight % highly cross-linked polymer blends, the Lava Ultimate has 80 weight % (65 vol%) nanoceramic particles (zirconia filler (4–11 nm), silica filler (20 nm), aggregated zirconia/silica cluster filler), 20 weight % (35 vol%) highly cross- linked (methacrylate-based) polymer matrix, and the Vita Enamic is composed of a 86 weight % feldspathic-based ceramic network and 14 weight % acrylate polymer network (infiltrated into feldspathic-based ceramic network). The first difference that emerged among the blocks is the low percentage of the matrix which is below 20% in the materials that did not change the surface roughness after toothbrushing. 

Koizumi et al. [21] tested six different “resin-ceramic” CAD/CAM materials such as Vita Enamic (Vita Zahnfabrik), Gradia Block (GC), Shofu Block HC (Shofu), Lava Ultimate (3M ESPE), Katana Avencia block (Kuraray Noritake Dental), and Cerasmart (GC) after simulating a toothbrushing of five years. The profilometer was used to detect the surface roughness. The results showed a significant difference, regarding the Ra, in the Cerasmart and Shofu Block HC materials after toothbrush abrasion compared with the control group represented by the ceramic (Vita Marks II, Vita Zahnfabrik). Also, these findings are conducible to the “nanofillers” type, not only to the inorganic filler contents but also filler size, filler form, and polymeric matrix [26]. Kamonkhantiku et al. [23] tested the surface roughness of six resin-contained CAD/CAM materials such as Shofu Block Hc (Shofu), Cerasmart (GC), Gradia Block (GC), Hybrid Resin, Block (Yamamoto), Lava Ultimate (3M, ESPE), and Vita Enamic (Vita Zahnfabrik) after 40,000 cycles of toothbrushing. The statistical analyses indicated that significant differences were found in Ra between the measuring stages for each material tested except for the Gradia Block (GC) and Vita Enamic (Vita Zahnfabrik). The authors attributed the differences in wear to the chemical compositions. The Gradia Block (GC) consists of large irregularly shaped silicate glass and numerous pre-polymerized filler particles that could possibly protect its soft resin matrix from toothbrushing, instead the Vita Enamic (Vita Zahnfabrik) is constructed with ceramic filler. However, the conclusions reported that all materials present an acceptable toothbrush wear resistance.

No relationships between toothbrushing and surface roughness (Ra) emerged in the study conducted by Mormon et al. [20]. The investigated samples include Lava Ultimate (3M ESPE),Vita Enamic (Vita Zahnfabrik), and other ceramic blocks such as zirconia and lithium disilicate. All the specimens were stored for 7 days in 37 °C deionized water, and successively were mounted in a toothbrushing machine for 40,000 cycles. However, the authors concluded that the experimental toothbrushing wear in the present study significantly reduced the gloss of enamel and of all material specimens, except zirconium dioxide ceramic. Instead, de Andrade et al. [24] determined significant differences among the chairside CAD-CAM materials and simulated toothbrushing. The authors submitted the sample to 100,000 brushing strokes, which simulated 10 years of clinical wear. The sample analyzed was composed of IPS Empress CAD (Ivoclar Vivadent AG), Cerasmart (GC), Vita Enamic (Vita Zahnfabrik), Lava Ultimate (3M, ESPE), and Grandio Block (VOCO GmbH). After brushing, the IPS Empress CAD (Ivoclar Vivadent AG) showed the lowest Ra values, followed by the Lava Ultimate (3M, ESPE) and the Vita Enamic (Vita Zahnfabrik). Instead, the other materials have the highest Ra values after brushing. Indeed, the Cerasmart (GC) and Grandio Block (VOCO GmbH) reached mean roughness values higher than the threshold Ra value of 0.2 μm reported in the literature [27].

Nima et al. [25] submitted ten specimens of Vita Enamic (Vita Zahnfabrik) and Lava Ultimate (3M, ESPE) to 300,000 toothbrushing strokes. The results showed an increase in roughness (Rv = maximum relative depth) and gloss before and after toothbrushing. Although all the articles examined used different toothpastes with no homogeneous RDA, different toothbrushing machine, and cycles of brushing, the findings are about the same. Some authors tested the resin-contained CAD/CAM materials from 40,000 cycles to 1500 cycles [20,21,22,23,24,25]. Koizumi et al. [21] brushed the specimens for 120 min (20,000 cycles). Assuming that the ideal time for toothbrushing is 120 s two times a day [28,29], the 20,000 cycles may correspond to an amount of five years. However, in literature the articles reported that the actual mean brushing time is 65.2 to 83.5 s per day [29]. Therefore, the studies may correspond to a clinical simulation with a range of 1 to 20 years. Regarding the different granulometry present in the toothpastes, the authors used different RDA index values in the experiments, which influenced the surface roughness of the resin-contained CAD/CAM materials investigated in the articles in the same ways [18,19,20,21,22,23].The reason for this comportment is attributable to the compositions of the resin-contained CAD/CAM materials. Indeed, blocks such as Lava Ultimate present 69% SiO_2_ and 31% ZrO_2_ fillers that improve the surface resistance to wear and the slight change in surface roughness after toothbrushing were considered clinically acceptable [21,22]. The aspect of the surface roughness remains a difficulty that clinicians do not consider. The literature reported 0.2 μm as the threshold value above which the plaque accumulation on dental materials increase [27]. However, it is difficult to measure the value clinically and no authors assessed the bristles’ effects on the materials. Therefore, a correct knowledge of the composition of dental material and the possible effect of toothbrushing is fundamental to obtain success and survival of the prosthetic rehabilitations. In summary, the main limitation encountered in the majority of the included studies consists of the assessment of resin-contained CAD/CAM material only in vitro studies without including the different clinical aspects such as saliva, blood, different types of beverages, and the daily comportment of patients. Other drawbacks of this systematic review have been the lack of studies in this field, however, the results of the articles highlighted the effect of toothbrushing on resin-contained CAD/CAM materials. New clinical and in vitro studies are needed to improve the dental hygiene of our patients and to prevent the increase of pathologies that correlate to plaque accumulation.

## 5. Conclusions

With the limitations of this study, we can conclude that the surface roughness of most resin-contained CAD/CAM materials was affected by artificial toothbrushing. Therefore, a correct knowledge of the composition of the dental material and toothpastes is fundamental to avoid an increase of surface roughness on the prosthetic rehabilitations. Moreover, future clinical studies are needed to assess the behavior of resin-contained CAD/CAM materials in clinic situations.

## Figures and Tables

**Table 1:** Data collection.

Authors	Year	Study Design	Sample	Toothbrushing Test	Surface Roughness Analysis	Roughness Parameter Measured	Correlation Toothbrushing and Surface Roughness
Morman et al. [20]	2013	In vitro	Lava Ultimate (3M ESPE) Vita Enamic (Vita Zahnfabrik)	Toothbrush Machine unspecified	Profilometer (Form Talysurf S2, Taylor Hobson, England).	Ra (μm)	NO
Koizumi et al. [21]	2015	In vitro	Vita Enamic (Vita Zahnfabrik) Gradia Block (GC) Shofu Block Hc (Shofu) Lava Ultimate (3M ESPE) Katana Avencia (Kuraray Noritake) Cerasmart (GC)	Toothbrush machine (K236, Tokyo Giken)	Profilometer (Surfcom 1400A, Tokyo Seimitsu, Tokyo, Japan)	Ra (μm) Rz (μm)	YES (Cerasmart and Shofu Block) NO (other materials)
Flury et al. [22]	2016	In vitro	Paradigm MZ100 (3M ESPE) Lava Ultimate (3M ESPE) Vita Enamic (Vita Zahnfabrik) Ambarino High Class (Creamed)	Toothbrush machine (Syndicad LR1)	Profilometer (Perthometer S2; Mahr GmbH)	Ra (μm) Rz (μm)	YES (Ambarino) NO (Lava Ultimate and Vita Enamic)
Kamonkhantikul et al. [23]	2016	In vitro	Shofu Block Hc (Shofu) Cerasmart (GC) Gradia Block (GC) Hybrid Resin Block (Yamamoto) Lava Ultimate (3M ESPE) Vita Enamic (Vita Zahnfabrik)	Toothbrush machine (V-8 Cross Brushing Machine, SABRI Dental)	Profilometer (Talyscan 150, Taylor Hobson, Leicester, England)	Ra (μm)	YES
Schmitt de Andrade et al. [24]	2021	In vitro	Cerasmart (GC) Vita Enamic (Vita Zahnfabrik) Lava Ultimate (3M ESPE)	Toothbrush machine (MEV2; Odeme Dental Research)	Contact profilometer (MaxSurf XT 20; Mahr).	Ra (μm)	YES
Nima et al. [25]	2021	In vitro	Vita Enamic (Vita Zahnfabrik) Lava Ultimate (3M ESPE)	Toothbrush machine (Maquina de Escivaca; Biopdi)	A 3D noncontact laser-scanning microscope (LEXT OLES4000 3D; Olympus).	Rv (μm)	YES

**Table 2:** Risk of bias assessment (ROBINS-I).

Study	Pre-Intervention		At Intervention	Post-Intervention				Overall Risk of Bias
	Confounding	Selection	Classification of Intervention	Deviation Fromintended Intervention	Missing Data	Measurement of Outcome	Reporting Result	
Morman et al. [20]	M	M	L	M	S	M	M	M
Koizumi et al. [21]	M	L	M	M	S	M	L	M
Flury et al. [22]	L	L	L	M	L	L	L	L
Kamonkhantikul et al. [23]	L	M	M	L	L	L	L	L
Schmitt de Andrade et al. [24]	L	M	L	M	L	L	L	L
Nima et al. [25]	L	M	M	L	L	L	L	L

L = “low risk of bias”; M = “moderate risk of bias”; S = “serious risk of bias”; C = “critical risk of bias”.

## Data Availability

Not applicable.
